# Estimating the prevalence of *Echinococcus* in domestic dogs in highly endemic for echinococcosis

**DOI:** 10.1186/s40249-018-0458-8

**Published:** 2018-08-09

**Authors:** Cong-Nuan Liu, Yang-Yang Xu, Angela M. Cadavid-Restrepo, Zhong-Zi Lou, Hong-Bin Yan, Li Li, Bao-Quan Fu, Darren J. Gray, Archie A. Clements, Tamsin S. Barnes, Gail M. Williams, Wan-Zhong Jia, Donald P. McManus, Yu-Rong Yang

**Affiliations:** 1 State Key Laboratory of Veterinary Etiological Biology/Key Laboratory of Veterinary Parasitology of Gansu Province/Key Laboratory of Zoonoses of Agriculture Ministry/Lanzhou Veterinary Research Institute, CAAS, Lanzhou, 730046 People’s Republic of China; 2 The Human Pathology and Immunology Department, Ningxia Medical University, Yinchuan, Ningxia Hui Autonomous Region People’s Republic of China; 3 Neurosurgery Department, Tianjin Xiqing Hospital, Tianjin, People’s Republic of China; 4 Molecular Parasitology Laboratory, QIMR Berghofer Medical Research Institute, Brisbane, Australia; 5 Research School of Population Health, Australian National University, Canberra, Australia; 6 School of Population Health, Infectious Disease Epidemiology Unit, University of Queensland, Brisbane, Australia; 7 Queensland Alliance for Agriculture and Food Innovation, University of Queensland, Gatton, Australia

**Keywords:** Domestic dog surveys, Dog-copro-multiplex PCR assay, Co-endemicity of *Echinococcus granulosus* and *E. multilocularis*, Xiji County, Ningxia hui autonomous region (NHAR), P. R. China

## Abstract

**Background:**

Cystic echinococcosis (CE) and alveolar echinococcosis (AE) are highly endemic in Xiji County of Ningxia Hui Autonomous Region (NHAR) in China where the control campaign based on dog de-worming with praziquantel has been undertaken over preceding decades. This study is to determine the current prevalence of *Echinococcus granulosus* and *E. multilocularis* in domestic dogs and monitor the echinococcosis transmission dynamics.

**Methods:**

Study villages were selected using landscape patterns (Geographic Information System, GIS) for *Echinococcus* transmission “hot spots”, combined with hospital records identifying risk areas for AE and CE. A survey of 750 domestic dogs, including copro-sampling and owner questionnaires, from 25 selected villages, was undertaken in 2012. A copro-multiplex PCR assay was used for the specific diagnosis of *E. granulosus* and *E. multilocularis* in the dogs. Data analysis, using IBM SPSS Statistics, was undertaken, to compare the prevalence of the two *Echinococcus* spp. in dogs between four geographical areas of Xiji by the *χ*^2^ test. Univariate analysis of the combinations of outcomes from the questionnaire and copro-PCR assay data was carried out to determine the significant risk factors for dog infection.

**Results:**

The highest de-worming rate of 84.0% was found in the northwest area of Xiji County, and significant differences (*P* <  0.05) in the de-worming rates among dogs from the four geographical areas of Xiji were detected. The highest prevalence (19.7%, 59/300) of *E. multilocularis* occurred in northwest Xiji, though the highest prevalence (18.1%, 38/210) of *E. granulosus* occurred in southwest Xiji. There was no significant difference (*P* >  0.05) in the prevalence of *E. granulosus* in dogs from the northwest, southwest, northeast, and southeast of Xiji, but there were significant differences (*P* <  0.05) between dogs infected with *E. multilocularis* from the four areas. None of the other independent variables was statistically significant.

**Conclusions:**

The results from this study indicate a high prevalence of both *E. granulosus* and *E. muiltilocularis* in dogs in Xiji County, NHAR. Transmission of *E. multilocularis* was more impacted by geographical risk-factors in Xiji County than that of *E. granulosus*. Dogs have the potential to maintain the transmission of both species of *Echinococcus* within local Xiji communities, and the current praziquantel dosing of dogs appears to be ineffective or poorly implemented in this area.

**Electronic supplementary material:**

The online version of this article (10.1186/s40249-018-0458-8) contains supplementary material, which is available to authorized users.

## Multilingual abstracts

Please see Additional file 1 for translations of the abstract into the five official working languages of the United Nations.

## Background

Human cystic echinococcosis (CE) and alveolar echinococcosis (AE), caused by the larval stages of *Echinococcus granulosus* and *E. multilocularis*, respectively, result from the unintentional ingestion of *Echinococcus* eggs released in the faeces of definitive hosts. Domestic dogs, and other suitable carnivores, are the usual definitive hosts of *E. granulosus*, whilst a number of ungulate species (goats, sheep, pigs, cattle, etc.) can act as intermediate hosts [1, 2]. Domestic dogs can also serve as definitive hosts of *E. multilocularis* if they become infected through the ingestion of small mammalian species (mainly rodents) infected with metacestodes, thus perpetuating a synanthropic cycle [3, 4]. Dogs become infected with *E. granulosus* after ingesting offal harbouring hydatid cysts containing viable protoscoleces. A sexually mature adult worm can develop from each protoscolex [5]. Depending on the species and strain (genotype), and on the susceptibility of the host, the adult tapeworm reaches sexual maturity approximately 4 to 6 weeks after infection [5]. Gravid proglottids or eggs are released in the feces and contaminate the external environment. Herbivores are usually exposed to infection from the pasture or from water supplies which may be contaminated by direct access of infected carnivores, where people and their domestic animals share drinking water, which is also accessible to dogs and/or wild animals [6, 7].

*E. granulosus* has a global distribution while *E. multilocularis* is confined to the northern hemisphere. It is well known that both *Echinococcus* spp. are of considerable public health significance, while *E. granulosus* causes substantial economic losses in husbandry/agriculture as has been reported in several provinces of the northwestern part of China [8]. Both CE and AE, are highly endemic in Xiji County, Ningxia Hui Autonomous Region (NHAR) where domestic and sylvatic lifecycles have been described [9, 10], which implies that the ecological environment there is conducive for transmission of both *Echinococcus* spp. The first investigations on the definitive dog and fox hosts for *E. granulosus* and *E. multilocularis* in Xiji were only reported about thirty years ago [11, 12]. A more recent investigation (2002–2003) there revealed that dog-ownership was one of the major risk factors for infection with both species of *Echinococcus* as shown by multiple regression analysis of the risk variables [13]. In order to curb the transmission of *E. granulosus* and *E. multilocularis*, the Chinese government is increasing financial support for echinococcosis control efforts [14], as despite the fact there have been extensive de-worming campaigns for domestic dogs launched by the government for preventing echinococcosis over the past decades, human exposure to *Echinococcus* infection remains at a high level [15, 16].

Surveillance data on canine *Echinococcus* infections are essential for establishing adequate intervention targets against CE and AE in hyper-endemic regions. This information may also help to monitor the transmission patterns of *Echinococcus* spp. based on their associations with local risk factors and the implementation of control measures. In addition, canine *Echinococcus* infections may also be used as an indicator of the potential risk of human infection [10, 17]. As part of an ongoing project of echinococcosis control in NHAR, we report on a survey of domestic dogs that was conducted in Xiji County in 2012, an area known to be highly endemic for both human AE and CE, to monitor the prevalence and transmission dynamics of *E. granulosus* and *E. multilocularis*.

## Methods

### Study area

Xiji County is located in the southern mountainous part of NHAR between latitudes 35°35′–36°14’ N, and between longitudes 105°20′–106°04 E. Xiji shares borders with three other NHAR counties: Haiyuan to the north, Guyuan to the east and Longde to the south, and with two counties from neighbouring Gansu Province, Huining and Jinning counties located to the west. The county has a total area of approximately 3985 km^2^ and is divided into three towns and 16 townships, comprising 306 administrative villages.

As with several other counties in the south of NHAR, Xiji is covered by rich forests and grasslands. The local landscape provides suitable habitats for a wide diversity of animal species. However, the natural environment there has been transformed in order to cultivate grain and other crops due to the increased human population which occurred during the 1980s and 1990s. Heavy land erosion, desertification and increased drought conditions have resulted in an echinococcosis pandemic in Xiji County [13, 17]. In addition, a reforestation campaign and the new “grain to green” policy were initiated at the beginning of this century [18]. Currently, several animal species that are suitable hosts for *Echinococcus* spp. are present in the area [18] and the resident number of dogs, including domestic and stray dogs, has been estimated to be similar in number to the human population in Xiji according to recent reports [19, 20].

### Dog survey design, copro-sampling and owner questionnaire

#### Design

GIS (Geographic Information Systems) technology was used in geo-morphological information searches to predict *E. multilocularis* transmission risk landscape profiles (‘hotspots’) [9] in terms of hosts assemblages (for suitable habitats) at high levels in order to select village communities for the dog survey. Village-community selection also used surgical records of human CE cases from hospitals [21] to predict locations of local *E. granulosus* transmission. As a result, a total of 25 rural villages in different towns within Xiji County were selected for the survey.

#### Questionnaire

Written consent was obtained from all dog-owners who agreed to participate in the survey, following an explanation of the benefits of the project for the local population. Then, local veterinary staff administered a short questionnaire to each dog-owner whose dog was involved in the survey. Demographic data recorded for each dog included: age, sex, skin-color and weight; whether categorized as a property guard dog, pet dog or herd guard dog; details of its roaming behaviour; previous de-worming history (date of last de-worming treatment, frequency of de-worming treatment, de-wormer used and dosage). The potential risk-factors recorded for dog infection included: (1) the number of dogs kept by a family; (2) which individuals were usually responsible for feeding dog(s); (3) whether a dog had been observed eating small mammals, killing a family livestock animal, had been given livestock viscera or captured wild mammals as food; (4) and how the faeces from the family dog had been disposed.

#### Copro-sampling

Fecal samples were collected from a total of 750 domestic dogs, the majority of which were weighed in the study villages. Approximately 25 g fecal sample per dog was collected, sealed in a ziplock sealable plastic bag, and each sample was labeled with the village and dog identification numbers and the date of collection. The samples were then transported to the Key Laboratory of Zoonoses of Agriculture Ministry, Lanzhou Veterinary Research Institute, Chinese Academy of Agricultural Sciences, Lanzhou, Gansu Province. The samples were frozen at − 80 °C for at least 7 days to kill any potentially infective *Echinococcus* eggs, so as to ensure maximum staff safety when processing the feces. The samples were then transferred to a − 20 °C freezer until required for further processing and copro-analysis.

### Dog copro-analysis

#### DNA extraction

About 20 g dog feces were placed in a 50 ml centrifuge tube, which was then filled with sodium chloride solution prior to copro-DNA extraction [22, 23]. The tube was vortexed until the fecal material was completely broken up and then the sample centrifuged at 1000×*g* for 5 min. Five hundred μl of the supernatant was transferred to a 2 ml centrifuge tube, 1.5 ml ddH_2_O added to dilute the solution, and the tube centrifuged at 12000×*g* for 10 min. The supernatant was carefully discarded and 200 μl ddH_2_O added to suspend the sediment for DNA extraction. Total genomic DNA was extracted using a TIANamp Stool DNA Kit (TIANGEN, Beijing, China) following the manufacturer’s instructions; after elution in 50 μl ddH_2_O, the DNA concentration was determined spectrophotometrically (Thermo, NanoDrop 2000, USA), and the DNA sample used in a multiplex PCR assay.

### Multiplex PCR assay

A multiplex PCR system was established for simultaneous detection of *E. granulosus* sensu stricto and *E. multilocularis* based on *nad*1 and *nad*5 genes of *E. granulosus* and *E. multilocularis*, respectively [24]. The specificity and sensitivity of the PCR and potential interference by inhibitors in the feces were determined before assay application [24]. Briefly, PCR amplification was performed in a 25 μl mixture containing 12.5 μl PCR mix (dNTPs 2.5 mM of each, 2.5 μl 10 × Ex*Taq* Buffer, MgSO_4_ 25 mM, 0.25 μl Ex*Taq* DNA polymerase 5 U/μl) (TaKaRa, Dalian, Liaoning), 4 μl of primer mix, 2 μl DNA template, and ddH_2_O added to 25 μl. DNA fragments were amplified using the following optimized thermos-cycling conditions: 95 °C/5 min for denaturation; 35 cycles of 94 °C /30 s, 55 °C /30 s, 72 °C /40 s; and 72 °C /10 min extension. For all the multiplex PCR assays, positive DNA (DNA templates of *E. granulosus and E. multilocularis*), and negative (no-DNA) controls were included [24]. PCR products were visualized by electrophoresis in a 1.5% agarose gel stained with ethidium bromide [24].

### Data management and statistical analysis

Results of the dog-owner questionnaire and dog copro-PCR were double-entered by two different researchers into database spreadsheets. Data analysis was undertaken using IBM SPSS Statistics version 20.0.0 (SPSS, Chicago, IL, USA). For the descriptive analysis, the prevalence of *E. granulosus* and *E. multilocularis* were expressed as percentages. Differences among groups were compared using the *χ*^2^ test. Prevalence of *E.* g*ranulosus* and *E. multilocularis* among the surveyed dogs were then compared between selected villages. The level of statistical significance was set at *P <* 0.05. Univariate regression analyses for the combinations of outcomes from the questionnaire and the copro-PCR analysis were conducted to determine the significant risk factor/s for dog-infection with either *E. multilocularis* or *E. granulosus* and co-infection for both species when *P* < 0.05.

## Results

### De-worming frequency in surveyed dogs

The dog-owner interviews indicated that the majority of the surveyed dogs (68.9%, 517/750) had been de-wormed each month (12 times per annum). Dog dosing coverage varied in Xiji with the lowest rate (24.7%) occurring in the southeast, followed by a rate of 67.8% in the northeast, a rate of 79.5% in the southwest, with the highest de-worming rate of 84.0% evident in the northwest area (Table 1), indicating a significant discrepancy in the de-worming rates (*P* < 0.05) among dogs between the four geographical areas.

**Table 1:** Prevalence of *Echinococcus granulosus* (E.g.) and *E. multilocularis* (*E.m*.) in surveyed domestic dogs and de-worming frequencies by geographic location of villages in towns of Xiji County, NHAR

Location	Town*	Dog	Infection rate determined by PCR				Annual de-worming frequency	
		No.	*E.m.* (%)	E.g. (%)	*E.m.*/E.g. (%)	Total (%)	< 6 times	6–12 times (%)
North-west	Jiqiang	120	27 (22.5)	24 (20.0)	4 (3.3)	55 (45.8)	2	116 (96.6)
	Xinyin	90	18 (20.0)	13 (14.4)	3 (3.3)	34 (37.8)	24	48 (53.3)
	Honyao	30	9 (30.0)	6 (20.0)	3 (10.0)	18 (60.0)	0	30 (100.0)
	Tianpin	60	5 (8.3)	7 (11.7)	0 (0)	12 (20.0)	0	58 (96.6)
Sub-total		300	59 (19.7)	50 (16.7)	10 (3.3)	119 (39.7)	26	252 (84.0)
South-west	Zhenhu	60	0 (0)	17 (28.3)	0 (0)	17 (28.3)	14	41 (68.3)
	Pingfen	90	3 (3.3)	13 (14.4)	3 (3.3)	19 (21.0)	1	79 (87.8)
	Xinpin	30	4 (13.3)	2 (6.7)	2 (6.7)	8 (26.6)	0	29 (96.6)
	Xitan	30	2 (6.7)	6 (20)	1 (3.3)	9930.0)	12	18 (60.0)
Sub-total		210	9 (4.3)	38 (18.1)	6 (2.9)	53 (25.2)	27	167 (79.5)
South-east	Xiaohe	60	3 (5.0)	8 (13.3)	0 (0)	11 (18.3)	23	6 (10.0)
	Shizi	30	6 (20.0)	6 (20.0)	2 (6.7)	14 (46.7)	0	30 (100.0)
	Xinlong	60	14 (23.3)	9 (15.0)	0 (0)	23 (38.3)	28	1 (1.6)
Sub-total		150	23 (15.3)	23 (15.3)	2 (1.3)	48 (32.0)	51	37 (24.7)
North-east	Piachen	30	3 (10.0)	6 (20.0)	0 (0)	9 (30.0)	0	30 (100.0)
	Baiya	30	5 (16.7)	1 (3.3)	0 (0)	6 (20.0)	27	1 (3.3)
	Shagou	30	7 (23.3)	6 (20.0)	2 (6.7)	15 (50.0)	0	30 (100)
Sub-total		90	15 (16.7)	13 (14.4)	2 (2.2)	30 (33.3)	27	61 (67.8)
Total		750	106 (14.1)	124 (16.5)	20 (2.7)	250 (33.3)	141	517 (68.9)

Notes: Daying, Datan, Tuanjie and Xiazhai villages are located in Jiqiang Town; Miaoercha Village, Chelugou Village, Baicheng Village are located in Xinyin Town; the village of Xiaochagou is located in Honyao Town; Tianpin Village and Maopin Village are located in Tianpin Town; Supu Village and Puyu Village are located in Zhenhu Town; Wangnao, Zhangwu and Jintang Villages are located in Pingfen Town; Yangcha Village is located in Xinpin Town; Linjiagou Village is located in Xitan Town; the villages of Hongquan and Langcha are located in Xiaohe Town; Xindian Village is located in Shizi Town; the villages of Daiduan and Xiafan are located in Xinlong Town; Xiapu Village is located in Piachen Town; Kufanggou Village is located in Baiya Town; Shagou Village is located in Shagou Town

### The distribution and prevalence of *E. granulosus* and *E. multilocularis* in dogs

**Fig. 1:**
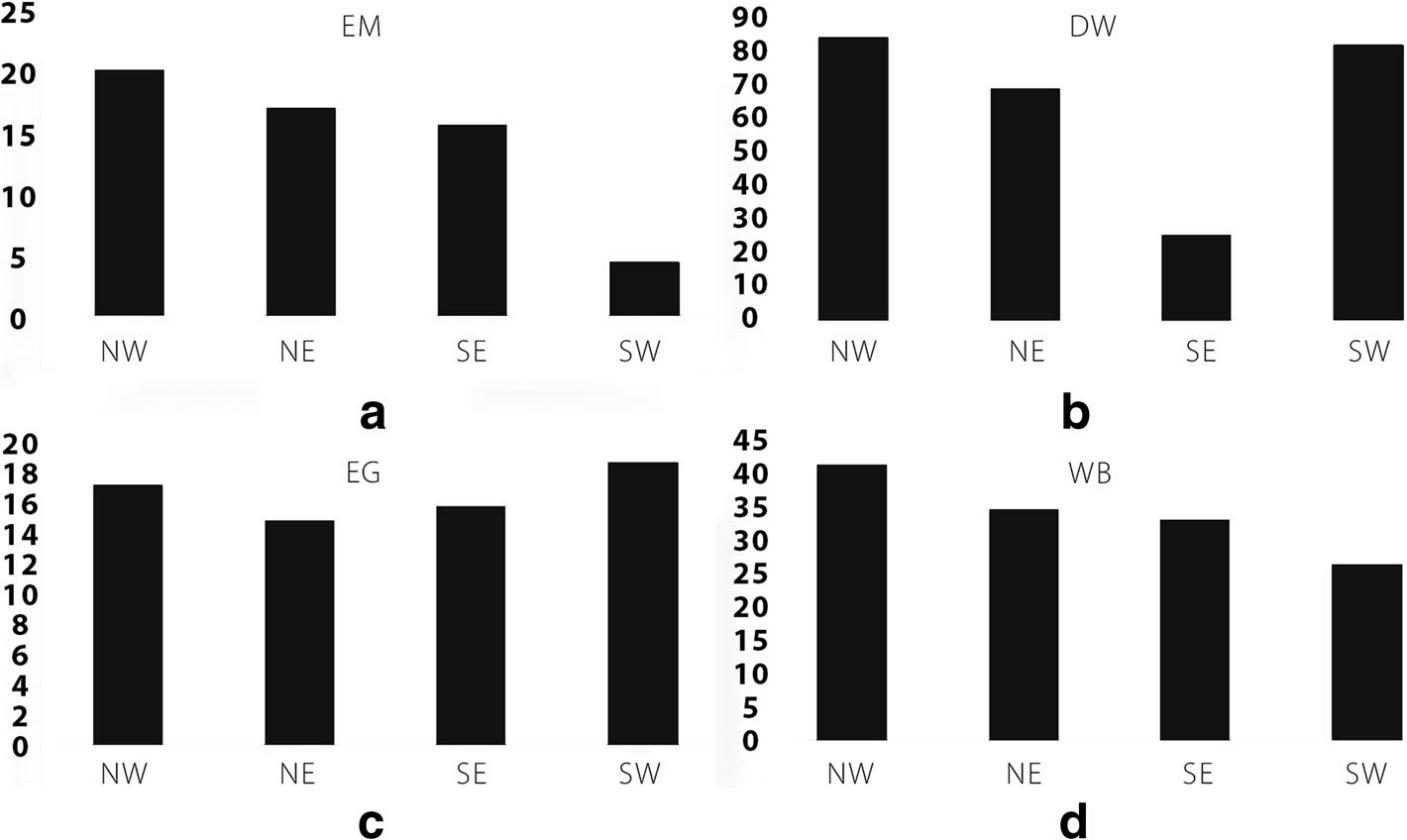
The prevalence of *E. granulosus* and *E. multilocularis* among domestic dogs. Panel **a**: Prevalence (y-axis) of *E. multilocularis*; Panel **b**: Prevalence (y-axis) of *E. granulosus*; Panel **c**: Total prevalence (y-axis) of *E. granulosus* and *E. multilocularis* and co-infections; Panel **d**: Percentage (y-axis) of dogs dewormed

The prevalence of infection with *E. granulosus* or *E. multilocularis* or co-infections with both species among the surveyed dogs, determined by the multiplex PCR, is summarized in Table 1. The highest prevalence (19.7%, 59/300) of *E. multilocularis* occurred in northwest Xiji, followed by 16.7% (15/90) in the northeast, 15.3% (23/150) in the southeast, and the lowest prevalence (4.3%, 9/210) occurred in the southwest; there was a significant difference in prevalence between the four geographical areas (*P* < 0.05). In direct contrast, the highest prevalence (18.1%, 38/210) of *E. granulosus* occurred in southwest Xiji, followed by 16.7, 15.3 and 14.4% in the northwest, in the southeast, and in the northeast, respectively, with no significant difference (*P* >  0.05) in prevalence among dogs evident between the four areas (Fig. 1). Only a small number of dogs (20) were found to be co-infected with *E. granulosus* and *E. multilocularis* (Table 1).

### Dog-infection risk factor analysis

The outcomes of univariate regression analysis for dog infection risk are summarized in Table 2. Of the surveyed dogs, 2/3 were adults and 2/3 were males. Most dogs were cared for by adult family members, the majority of families kept dogs for guarding property or livestock, and these were tied in the day-time but untied at night (where they were allowed to roam in the house-hold yard or around livestock pens, as indicated by some villagers after interview) although most of the interview (formal and publically answered questions) records showed that 95.2% (714/750) dogs were tied continually in the house-hold yard. Most families (3/4), on average, kept one dog, 85.5% of families (641/750) used dog-feces as farmland fertilizer, and 87.8% families (517 + 141/750) carried out dog de-worming (Table 1).

**Table 2:** Uni-variate regression analysis of dog-infection risk factors for *E. multilocularis* and *E. granulosus*

Variable	Description of variable	*E.m.*		E.g.	
	(*E.m.*/E.g.) Total dog number	*OR* (95% *CI*)	*P*-value	*OR* (95% *CI*)	*P*-value
Age	1–5/≥6 years. (89/93) 615/(13/18) 110	1.263 (0.679–2.349)	> 0.05	0.911 (0.525–1.580)	> 0.05
Sex	male/female (70/85) 507/(36/39) 242	0.917 (0.593–1.416)	> 0.05	1.048 (0.693–1.587)	> 0.05
Weight	≤ 20/> 20 kg (80/96) 571/(16/20) 147	1.334 (0.754–2.360)	> 0.05	1.283 (0.763–2.159)	> 0.05
Skin color	dark/brown/white/yellow/mix (46/34)265/(19/25)104/(21/31)146/(15/19)133/(5/14)99	–	> 0.05	–	> 0.05
How maintained	tied/allowed to roam (102/115) 714/(4/8) 36	1.333 (0.462–3.850)	> 0.05	0.672 (0.299–1.512)	> 0.05
Person feeding dog	adult/child+adult (78/80) 549/(24/37) 185	1.111 (0.680–1.816)	> 0.05	0.682 (0.443–1.050)	> 0.05
Dog number/family	1 dog/2–3 dogs (54/67) 358/(14/16) 102	1.117 (0.592–2.105)	> 0.05	1.238 (0.682–2.246)	> 0.05
Feed dog with offal	often/never (37/41) 215/(60/65) 440	1.317 (0.842–2.058)	> 0.05	1.360 (0.884–2.090)	> 0.05
Dogs seen eating small mammals	yes /no (15/11) 105/(68/73) 453	0.944 (0.516–1.727)	> 0.05	0.610 (0.311–1.194)	> 0.05
Home animal slaughter	often /never (63/70) 422/(40/42) 251	0.926 (0.602–1.425)	> 0.05	0.990 (0.651–1.505)	> 0.05
Buy meat	often/never (76/80) 492/(27/33) 181	1.042 (0.647–1.678)	> 0.05	0.871 (0.557–1.362)	> 0.05
Dog role	pet /guard-dogs^ (20/13) 89/(82/105) 642	1.980 (1.143–3.428)	< 0.05*	0.875 (0.469–1.633)	> 0.05
Feces used	fertilizer/placed in rubbish area (85/107) 641/(14/10) 71	0.622 (0.332–1.166)	> 0.05	1.222 (0.607–2.462)	> 0.05
De-worming	yes/no (84/105) 609/(19/13) 102	0.699 (0.404–1.210)	> 0.05	1.587 (0.858–2.935)	> 0.05

^ Guard-dogs, guard including both of family properties and family livestock (which is herd-guard); * Significant *P*-value

Guard-dogs had a significant association with the risk of dog infection with *E. multilocularis*. None of the other independent variables included in the analysis were statistically significant. Only 14% (105/750) dog-owners reported having observed their dogs eating small mammals.

## Discussion

The findings of this study provide a better understanding of the distribution and prevalence of *E. granulosus* and *E. multilocularis* among domestic dogs in Xiji County, NHAR, P. R. China. Reports to date on canine infections of *Echinococcus* spp. in NHAR are few [13, 18], although human case prevalence and incidence have been described in numerous publications [15, 17, 21, 25]. A high rate of exposure to *Echinococcus* eggs among children in Xiji County indicated very active transmission of both *E. granulosus and E. multilocularis* [15, 16], and the combined prevalence (33.3%) among the surveyed dogs determined in this study was very high. *E. multilocularis* transmission has been shown to be intense from the north-west to the eastern mountainous areas of NHAR, but much less transmission occurs on the southwest plateau area where few susceptible small mammals have been reported [9, 26] due to the unsuitable environment there [27]. These results verified our GIS analysis which was able to correctly predict areas of high and the low transmission risk. Further, this study provided evidence that active transmission of *E. multilocularis* occurred in some locations hyper-contaminated with eggs, where the sero-prevalence of human exposure (in school children, 6–18 years) was higher than in other areas (data will be published elsewhere). However, although *E. granulosus* transmission is more pronounced and the highest human prevalence has been recorded in the southwest plateau area compared with the other three mountainous areas [13, 25], the prevalence of *E. granulosus* among dogs was similar in the four locations. This can be explained by the increased trading of meat (lamb, mutton, beef) throughout the whole of Xiji, with more active trading in the southwest due to the completion of a new vehicle highway connecting the centre of Xiji County to the neighbouring province of Gansu via Huining County in Gansu [18]. The present study also provides evidence that current environmental contamination with *E. granulosus* eggs is far higher than reported about a decade ago when only a very small number of dogs were present in rural villages in Xiji due to many having been poisoned by a rodenticide which was used extensively in rodent-control campaigns [13]. The current high *E. granulosus* prevalence among dogs in most parts of Xiji is likely due to the abundance of viscera or carcasses, harbouring viable hydatid lesions, that can be accessed frequently and readily by domestic dogs in rural villages [18]. Furthermore, dogs would have more opportunity to eat small mammals infected with *E. multilocularis* larvae in those areas where GIS surveys predicted high numbers of susceptible rodent species [26]. The questionnaire data from the current study provides confirmatory evidence that dog owners observed dogs capturing small rodents as prey. Additionally, many rural families are less likely to provide nutrient-rich food to dogs. Therefore, undernourished dogs in rural areas, particularly guard-dogs that were allowed to roam at night, may be more pre-disposed to hunting which increases the risk of exposure to *Echinococcus* infection. Local villagers in NHAR habitually feed dogs with captured small mammals such as *Microtus fortis*, *Myospalax fontanieri*, *Meriones unguiculatus*, *Spermophilus dauricus* and *S. alashanicus* [13], most being susceptible hosts for *E. multilocularis*. Thus, it is likely that some of the captured mammals that were fed to dogs by villagers might harbor metacestodes of *E. multilocularis* resulting in the canines becoming infected.

Although it could be assumed that there should be lower worm infection in adult dogs compared with younger animals who have not yet acquired any immunity [28], the fact that more adult dogs were sampled than young dogs in this study could explain the relatively higher prevalence in the former (Table 2). Additionally, adult dogs were more pre-disposed to infection as they were more likely to feed on raw infected animal carcasses. A further factor was the increased opportunity to become infected due to their being allowed to roam freely when guarding property or livestock. These factors are well recognized as important infection risks for *E. granulosus* [29] and *E. multilocularis* [30] in dogs.

The very active factors for transmission of *E. multilocularis* and *E. granulosus* revealed here imply that previously reported factors, such as poor socio-economic situation, inadequate hygiene awareness, traditional lifestyles and animal husbandry practices, insufficient hygiene and close contact with dogs, had not yet been thoroughly eliminated [25], although a series of *Echinococcus* control campaigns have been launched by the Chinese government in echinococcosis-endemic regions and provinces of northwestern China [14]. In the China Government’s program for *Echinococcus* transmission control, one of the main intervention options is to use the drug praziquantel (PZQ) for dog de-worming. This is based on the fact that egg production in *E. granulosus* ranges from 34 to 58 days post-infection (but may vary according to the genotype of the parasite and/or the breed of dog infected), whereas in *E. multilocularis* egg production commences 28–35 days post-infection. Thus, monthly deworming with PZQ were carried out for *E. granulosus* and for *E. multilocularis* in areas where the two species co-exist [31, 32].

Praziquantel is provided through the local Animal Centre of Disease Control (ACDC) to dog-owners each month for administration of their dogs with a dose of 5 mg/kg [33] by the control project support team.

Despite the extensive control efforts though dog de-worming monthly, there was no correlation between the *Echinococcus* spp. prevalence among the dogs and the frequencies of PZQ de-worming in areas of Xiji County, which stimulated our investigation of the causes for this anomaly. The high level of *Echinococcus* infection among the studied dogs suggests that once per month PZQ de-worming did not effectively control transmission as it can be assumed that, after 24–48 h, the effect of the drug would cease, and the dogs are immediately susceptible to a new infection. Furthermore, we did not obtain all the dog faeces for copro-testing prior to and post de-worming. Due to the lack of DNA test data for de-worming, this study could not provide accurate evidence to assess the deworming effectiveness/inefficiency to guide the control program. Dogs are highly susceptible to infection by both *E. multilocularis* and *E. granulosus* [34]. Therefore, particular attention should be paid to the treatment of domestic canines from poor rural areas so as to aid in the control of echinococcosis and prevent transmission. Accurate diagnosis of *Echinococcus* spp. infections in definitive hosts plays a central role in the surveillance of echinococcosis control programs both for establishing baseline data at the commencement of the program and for monitoring its effectiveness. It can also provide an indicator of the potential risk to humans of being infected.

The high *Echinococcus* prevalence in dogs in the study area and the dispersal and transmission of the parasites [10, 15, 18] may be partially explained by a number of factors which include: ready access by dogs to infected small mammals with *E. multilocularis,* and animal viscera, offal or carcasses with hydatid cysts of *E. granulosus*; inefficient or ineffective de-worming of dogs with PZQ and/or poor treatment compliance by dog owners; and the absence of regular inspections of dogs for the presence of the tapeworms. It is vital to continually update epidemiological and infection data on domestic dogs, so as to assist in the design of a cost-effective prevention and control program for human echinococcosis in southern NHAR and other endemic regions in China and elsewhere. A health education strategy in concert with knowledge of the educational background, culture and religion of the local population will be a key feature to effectively disseminate knowledge on the importance of dogs in the transmission of human AE and CE in order to improve key attitudes and behaviours leading to effective control in the future.

## Conclusions

The paper describes the findings on *E. multilocularis* and *E. granulosus* transmission maintained by dogs after a long-term dog de-worming control program in Xiji County, China, and investigated the *Echinococcus* prevalence in domestic dogs in this community highly endemic for echinococcosis. The investigation revealed that dog infection rates were high for both *E. multilocularis* and *E. granulosus*, indicating the current dog dosing regimen appears to be ineffective or is unsuitably implemented in this study area.

## Additional file

Additional file 1: Multilingual abstracts in the five official working languages of the United Nations. (PDF 514 kb)

## Abbreviations

AE: Alveolar echinococcosis
CE: Cystic echinococcosis
GIS: Geographic information systems
NHAR: Ningxia Hui Autonomous Region
PCR: Polymerase chain reaction
PZQ: Praziquantel
SPSS: Statistical package for social sciences
