# Supplementary material for: Estimating the prevalence of Echinococcus in domestic dogs in highly endemic for echinococcosis
Source: Infect Dis Poverty. 2018 Aug 9;7:77. doi: 10.1186/s40249-018-0458-8 (PMC6083587; doi:10.1186/s40249-018-0458-8)
Supplement: Supplementary file 1 — Multilingual abstracts in the five official working languages of the United Nations. (PDF 514 kb) [file 40249_2018_458_MOESM1_ESM.pdf]

Translation of the abstract into the five official working languages of the United Nations

انتشار داء المشوِكة في الكلاب المنزلية بالصين المستوطنة لداء المشوكات

كونغ-نوان ليو، يانغ-يانغ كسو، آنجيلا م. كادافيد-ريستريبو، زونغ-زي لو، هونغ-بِن يان، لي لي، باو-كوان فو، دارِن جي. غراي، آرتشي آي. كليمينتس، تامسِن س. بارنز، غايل م. ويليامز، وان-زونغ جيا، دونالد ب. ماك مانوس، و يو-رونغ يانغ

الملخص

الخلفية: إن داء المشوِكات الكيسية (AE) والمشوكات السنخية (CE) مستوطنان بشكل كبير في مقاطعة زيجي من منطقة نينغشيا ذات الحكم المستقل لقومية هوي (NHAR) في الصين حيث ما زالت حملة السيطرة على هذا الداء، من خلال مكافحة الديدان في الكلاب باستخدام برازيكوانتيل، مستمرة منذ عقود مضت. تهدف هذه الدراسة إلى تحديد مدى الانتشار الحالي للمشوِكة الحُبيبية والمشوِكة عديدة المساكن بين الكلاب المنزلية ومراقبة ديناميكيات انتقال داء المشوكة.

الطرق: تم اختيار قرى الدراسة باستخدام أنماط المناظر الطبيعية (نظام المعلومات الجغرافي، GIS) لإيجاد بؤر نشاط المشوِكة عديدة المساكن، بالإضافة إلى دراسة السجلات الطبية للتعرف على مناطق الخطر بسبب المشوِكة الكيسية والمشوِكة السنخية. أجريت في عام 2012 دراسة استقصائية شملت 750 كلبا منزلياً، وتضمنت هذه الدراسة جمع عينات من البراز بالإضافة إلى استبيانات موجهة لمالكي هذه الكلاب، والذين ينتمون إلى 25 قرية مختارة. تم استخدام التحليل المتعدد للبراز للتشخيص المحدد للمشوِكة الحُبيبية و المشوِكة عديدة المساكن بين تلك الكلاب. تم تحليل البيانات باستخدام نظام تحليل البيانات الإحصائية IBM SPSS، وذلك بهدف مقارنة انتشار نوعي المشوكة في الكلاب بين أربع مناطق جغرافية من مقاطعة زيجي من خلال استخدام اختبار χ 2. تم إجراء تحليل أحادي المتغير للتركيبات الناتجة عن نتائج الاستبيان بالإضافة إلى فحص البيانات الناتجة عن تحليل البراز وذلك بهدف تحديد عوامل الخطر ذات الدلالة المتعلقة بحدوث العدوى عند الكلاب.

النتائج: توصل الباحثون إلى أن أعلى نسبة لمكافحة الديدان هي 84% وذلك في المنطقة الشمالية الغربية من مقاطعة زيجي، حيث وجدت اختلافات ذات دلالة (0.05 < P) في نسب مكافحة الديدان في الكلاب وذلك في الأربع مناطق الجغرافية من مقاطعة زيجي. أوسع انتشار للدودة المشوِكة الحبيبية بنسبة (18.1%،210/38) وجد في جنوب غرب مقاطعة زيجي، بينما وجد أوسع انتشار (19.7%, 300/59) للدودة المشوِكة عديدة المساكن في شمال غرب زيجي. لم يكن هناك أية اختلافات ذات دلالة (0.05 < P) في انتشار الدودة المشوِكة الحُبيبية عند الكلاب في الشمال الغربي، والجنوب الغربي، والشمال الشرقي، والجنوب الغربي من مقاطعة زيجي، ولكن وجدت اختلافات ذات دلالة (0.05 < P) بين الكلاب المصابة بالدودة المشوِكة عديدة المساكن في المناطق الجغرافية الأربع الآنف ذكرها. ولم تكن لأيٍ من المتغيرات المستقلة الأخرى أي دلالة إحصائية.

الاستنتاجات: تشير نتائج هذه الدراسة إلى انتشار مرتفع لكلا الدودتين المشوِكة الحُبيبية والمشوِكة عديدة المساكن عند الكلاب في مقاطعة زيجي الواقعة في منطقة نينغشيا ذات الحكم المستقل لقومية هوي. وكان انتقال الدودة المشوِكة عديدة المساكن أكثر تأثراً بعوامل الخطر الجغرافية في مقاطعة زيجي من الدودة المشوكة الحُبيبية. لدى الكلاب القدرة على إبقاء انتقال كلا النوعين من الدودة المشوِكة ضمن مجتمعات مقاطعة زيجي المحلية، وأما مكافحة الديدان باستخدام برازيكوانتيل فيبدو أنها غير فعالة أو أنها مطبقة بشكل سيء في تلك المنطقة.

Translated from English version into Arabic by Isra Alqudah and Free bird, through

TRANSLATORS
WITHOUT BORDERS

棘球蚴病高发地区家犬棘球绦虫感染率监测分析

Cong-Nuan Liu, Yang-Yang Xu, Angela M. Cadavid-Restrepo, Zhong-Zi Lou, Hong-Bin Yan, Li

Li, Bao-Quan Fu, Darren J. Gray, Archie A. Clements, Tamsin S. Barnes, Gail M. Williams, Wan-Zhong Jia, Donald P. McManus and Yu-Rong Yang

引言：中国宁夏回族自治区西吉县是多房包虫病（AE）和单房包虫病（CE）的高流行区，基于犬用吡喹酮驱虫的控制活动已经在该县开展了几十年。本研究旨在确定目前家犬中的细粒棘球绦虫和多房棘球绦虫的流行情况，并对棘球绦虫病的传播动力学进行探究。
方法：利用空间分析法（地理信息系统），结合医院临床 AE 和 CE 病例记录,选择棘球蚴病的流行的“热点”村为调查对象。2012 年，对来自 25 个选定村庄的 750 只家犬进行了犬粪检测和犬主问卷调查，采用针对犬粪检测研发的多重 PCR 方法对犬的细粒棘球绦虫和多房棘球绦虫感染进行特异诊断，IBM SPSS 统计软件分析比较两种棘球绦虫的感染率。在西吉县的西北、东北、西南和东南四个地理区域之间进行 χ^2 检验。对问卷结果和犬粪 PCR 检测数据结合进行单变量分析以确定造成犬感染有显著意义的危险因素。
结果：西吉县四个区域犬的驱虫率有显着差异（$P < 0.05$），其中西北区域的驱虫率最高，为 84.0%，但西北部犬多房棘球绦虫感染率最高（19.7%，59/300）。虽然犬单房棘球绦虫最高感染率 18.1％（38/210）发生在西南区域，全县四个区域（西北，西南，东北和东南）犬的细粒（单房）棘球绦虫感染率无显著差异（$P > 0.05$），但多房棘球绦虫感染率在四个区域中差异显著（$P < 0.05$）。研究中的其他统计分析指标在四个区域中均未见显著差异。
结论：本研究结果表明，细粒棘球绦虫和多房棘球绦虫在宁夏西吉县犬中的感染率很高。地理风险因素对多房棘球绦虫传播的影响要大于细粒棘球绦虫。犬应该是两种棘球绦虫在西吉各地区内传播的主要宿主，目前吡喹酮在犬体内的驱虫剂量似乎无效或驱虫工作在西吉县各区域实施得不理想。

Translated from English version into Chinese by Cong-Nuan Liu

La prévalence de l'*Echinococcus* chez les chiens domestiques dans un environnement infecté par l'échinococcose hautement endémique en Chine.

Cong-Nuan Liu, Yang-Yang Xu, Angela M. Cadavid-Restrepo, Zhong-Zi Lou, Hong-Bin Yan, Li Li, Bao-Quan Fu, Darren J. Gray, Archie A. Clements, Tamsin S. Barnes, Gail M. Williams, Wan-Zhong Jia, Donald P. McManus et Yu-Rong Yang.

Résumé
Contexte : L’échinococcose kystique (AE) et l’échinococcose alvéolaire (CE) sont hautement endémiques dans le comté de Xiji, situé dans la région autonome Hui du Ningxia, en Chine. La campagne de contrôle, qui dure depuis les dernières décennies, repose sur le traitement vermifuge des chiens au praziquantel. Cette étude cherche à déterminer la prévalence actuelle de *l'Echinococcus granulosus* et de*l'Echinococcus multilocularis* chez les chiens domestiques et à surveiller la dynamique de transmission de l’échinococcose.
Méthodes : Les villages à l’étude ont été choisis selon certaines tendances sur le terrain (provenant du système d’informations géographiques, GIS) liées aux points chauds de la transmission de l’Echinococcus multilocularis, et selon des dossiers médicaux qui établissent des zones à risques d’AE et de CE. Depuis 2012, nous avons étudié 750 chiens domestiques dans 25 villages sélectionnés, en incluant un échantillonnage et l’interrogation des propriétaires, entre autres. Un

essai PCR copromultiplex a été utilisé pour obtenir un diagnostique spécifique à *l'Echinococcus granulosus* et à *l'Echinococcus multilocularis* chez les chiens. Des analyses de données, effectuées avec l'aide des statistiques d'IBM SPSS et d'un test χ^2 ont été faites, pour comparer la prévalence des deux Echinococcus spp chez les chiens entre quatre territoires géographiques du Xiji. Une analyse univariée des résultats combinés provenant des questionnaires et des données d'essai copro PCR a été effectué pour déterminer les facteurs de risques significatifs des infections canines.

Résultats : Le taux le plus élevé de vermifugation, à 84,0 %, se situe dans la région nord-ouest du comté de Xiji, et des différences significatives (P < 0,05) ont été constatées entre les taux de vermifugation de chiens de quatre régions géographiques du Xiji. La prévalence d'*Echinococcus granulosus* la plus élevée (18,1 %, 38/210) s'est produite dans la région sud-ouest du Xiji, tandis que la prévalence d'*Echinococcus multilocularis* la plus élevée (19,7 %, 59/300) s'est produite dans la région nord-ouest du Xiji. Il n'existait aucune différence significative (P > 0,05) dans la prévalence de l'Echinococcus granulosus chez les chiens situés dans les régions nord-ouest, sud-ouest, nord-est et sud-est du Xiji. Mais il existait des différences significatives (P < 0,05) entre les chiens infectés par l'Echinococcus multilocularis dans ces quatre régions. Aucune des autres variables indépendantes n'était statistiquement significative.

Conclusions : Les résultats de cette étude indiquent une prévalence élevée de l'Echinococcus granulosus et de l'Echinococcus multilocularis chez les chiens du comté de Xiji, dans la région autonome Hui du Ningxia. La transmission de l'Echinococcus multilocularis dépendait davantage des facteurs de risques géographiques dans le comté de Xiji que l'Echinococcus granulosus. Les chiens peuvent potentiellement maintenir le niveau de transmission des deux types d'Echinococcus dans les communautés du Xiji, et le dosage de praziquantel actuel donné aux chiens semble inefficace ou ne semble pas être mis en place de façon systématique dans cette région.

Translated from English version into French by Nathalie Thompson and Chloé Léger, through

TRANSLATORS
WITHOUT BORDERS

Распространённость *эхинококка* среди домашних собак в высоко эндемичном по эхинококкозу Китае

Цун-Нуань Лю, Ян-Ян Сюй, Анджела М. Кадавид-Рестрепо, Чжун-Цзы Лоу, Хун-Бинь Янь, Ли Ли, Бао-Цюань Фу, Даррен Дж. Грей, Арчи А. Клементс, Тасмин С. Барнс , Гэйл М. Уильямс, Вань-Чжун Цзя, Дональд П. Макманус и Юй-Жун Ян

Аннотация

Справочная информация: Кистозный эхинококкоз (КЭ) и альвеолярный эхинококкоз (АЭ) являются высоко эндемичными заболеваниями в уезде Сицзи Нинся-Хуэйского автономного района Китая, где в последние десятилетия проводится кампания по контролю заболеваемости, основанная на дегельминтизации собак с применением празиквантела. Целью настоящего исследования является определить текущую распространённость

инфекций *Echinococcus granulosus* и *E. multilocularis* среди домашних собак, а также проследить динамику передачи эхинококкоза.

Методы: Деревни для обследования были отобраны по структуре ландшафта (Геоинформационная система, ГИС) согласно «горячим точкам» передачи E. multilocularis в сочетании с больничными записями, указывающими на критические области по КЭ и АЭ. В 2012 году в 25 отобранных деревнях было проведено обследование 750 домашних собак, в том числе выборка по копрограмме и анкетирование хозяев. Для специфической диагностики инфекций *E. granulosus* и *E. multilocularis* у собак были взяты пробы на анализ ПЦР по комплексной копрограмме. Для сравнения распространённости двух видов эхинококка у собак в четырёх географических районах Сицзи, был проведён анализ данных по критерию χ^2 («хи квадрат») с использованием компьютерной программы IBM SPSS Statistics. Определение существенных факторов риска инфицирования собак производилось при помощи однофакторного анализа по сочетанию итоговых данных анкетирования и анализа проб ПЦР по копрограмме.

Результаты: Наиболее высокий уровень дегельминтизации 84,0% был обнаружен в северо-западной волости уезда Сицзи, при этом во всех четырёх географических волостях уезда Сицзи наблюдались существенные различия ($P < 0,05$) по уровню дегельминтизации собак. Самая высокая распространённость (18,1%, 38/210) инфицирования *E. granulosus* наблюдалась на юго-западе Сицзи, тогда как наивысшее значение показателя распространённости (19,7%, 59/300) инфекции *E. multilocularis* было зарегистрировано на северо-западе Сицзи. Между северо-западной, юго-западной, северо-восточной и юго-восточной волостями уезда Сицзи не наблюдалось существенной разницы в распространённости E. granulosus у собак ($P > 0,05$), однако в отношении заражения собак E. multilocularis были обнаружены значительные различия между четырьмя означенными регионами ($P < 0,05$). Ни одна из других независимых переменных не обладала статистической значимостью.

Выводы: Результаты данного исследования указывают на высокую распространённость как E. granulosus так и E. muiltilocularis у собак уезда Сицзи Нинся-Хуэйского автономного района. Передача E. multilocularis в уезде Сицзи была более обусловлена географическими факторами риска, чем заражение E. granulosus. Собаки обладают потенциалом поддерживать передачу обоих видов эхинококка среди местных сообществ уезда Сицзи, а предписываемая в настоящее время дозировка празиквантела для собак, по всей вероятности, является либо неэффективной, либо плохо реализуемой в указанном регионе.

Translated from English version into Russian by Liudmila Tomanek and Lisazaveta Frolova, through

TRANSLATORS
WITHOUT BORDERS

Prevalencia de *Equinococo* en perros domésticos en China en casos altamente endémicos de equinococosis

Cong-Nuan Liu, Yang-Yang Xu, Angela M. Cadavid-Restrepo, Zhong-Zi Lou, Hong-Bin Yan, Li Li, Bao-Quan Fu, Darren J. Gray, Archie A. Clements, Tamsin S. Barnes, Gail M. Williams, Wan-Zhong Jia, Donald P. McManus and Yu-Rong Yang

Resumen

Antecedentes: La equinococosis quística (EQ) y la equinococosis alveolar (EA) son altamente endémicas en el condado de Xiji de la región autónoma de Ningxia Hui (NHAR) en China, donde la campaña de control basada en la desparasitación canina con praziquantel se ha llevado a cabo durante las décadas anteriores. El objetivo de este estudio es determinar la prevalencia actual de *Equinococo granuloso* y *E. multilocularis* en perros domésticos y observar la dinámica de transmisión de la equinococosis.

Métodos: Los pueblos de estudio fueron seleccionados según patrones de paisaje (Sistema de Información Geográfica, SIG) para los "puntos críticos" de transmisión de E. multilocularis, combinados con registros hospitalarios que identifican áreas de riesgo para EA y EQ. En 2012 se llevó a cabo una encuesta sobre 750 perros domésticos, incluyendo copro muestreos y cuestionarios a propietarios, de 25 pueblos seleccionados. Se utilizó un ensayo de PCR copro-multiplex para el diagnóstico específico de *E. granuloso* y *E. multilocularis* en los perros. Se realizó un análisis de datos utilizando IBM SPSS Statistics para comparar la prevalencia de las dos especies de Equinococo en perros de cuatro áreas geográficas de Xiji mediante la prueba χ^2 . Se realizó un análisis univariado de las combinaciones de los resultados del cuestionario y de los datos de la prueba de copro-PCR para determinar los factores de riesgo significativos para la infección canina.

Resultados: La mayor tasa de desparasitación del 84,0% se encontró en el área noroeste del condado de Xiji, y se detectaron diferencias significativas ($P < 0,05$) en las tasas de desparasitación entre los perros de las cuatro áreas geográficas de Xiji. La prevalencia más alta (18,1%, 38/210) de *E. granuloso* ocurrió en el suroeste de Xiji, mientras que la prevalencia más alta (19,7%, 59/300) de *E. multilocularis* ocurrió en el noroeste de Xiji. No hubo diferencias significativas ($P > 0.05$) en la prevalencia de E. granulosus en perros del noroeste, suroeste, noreste y sureste de Xiji, pero sí hubo diferencias significativas ($P < 0.05$) entre perros infectados con E. multilocularis de las cuatro áreas. Ninguna de las otras variables independientes fue estadísticamente significativa.

Conclusión: Los resultados de este estudio indican una alta prevalencia de E. granuloso y E. muiltilocularis en perros del condado de Xiji, NHAR. La transmisión de E. multilocularis se vio más afectada por factores geográficos de riesgo en el condado de Xiji que la de E. granuloso. Los perros tienen el potencial de mantener la transmisión de ambas especies de Equinococo dentro de las comunidades locales de Xiji, y la dosis actual de praziquantel en perros parece ser ineficaz o mal implementada en esta área.

Translated from English version into Spanish by Pat Clivio and Xinia Arias, through

TRANSLATORS
WITHOUT BORDERS
